# Comparative Analysis of Maxillary Sinus Volume in Patients With Cleft Lip and Palate Versus Class III Malocclusion Patients Using CBCT

**DOI:** 10.1111/ocr.70041

**Published:** 2025-10-10

**Authors:** Sara Eslami, Anand Marya, Babak Sayahpour, Sarah Bühling, Stefan Kopp, Hanieh Mahmoudi, Ahmadreza Talaeipour, Ari Harsoputranto, Abdolreza Jamilian

**Affiliations:** ^1^ Department of Orthodontics, Center for Dentistry and Oral Medicine (Carolinum) Goethe University Frankfurt Frankfurt Germany; ^2^ Orthodontic department, Faculty of Dentistry University of Puthisastra Phnom Penh Cambodia; ^3^ The City of London Dental School University of Greater Manchester Bolton UK; ^4^ Faculty of Dentistry, Tehran Medical Science Islamic Azad University Tehran Iran; ^5^ Maxillofacial Radiology Department, Faculty of Dentistry, Tehran Medical Science Islamic Azad University Tehran Iran; ^6^ Orthodontic Department, Faculty of Dentistry, Tehran Medical Sciences Islamic Azad University Tehran Iran

## Abstract

**Objectives:**

This study compared maxillary sinus volume (MSV) in patients with unilateral non‐syndromic cleft lip and palate (CLP) to a skeletally matched control group with a skeletal class III pattern (group CTR) using cone‐beam computed tomography (CBCT) images.

**Materials/Methods:**

Ninety CBCT images were evaluated, including 45 patients with unilateral CLP (group CLP, mean age 24.3 ± 6.1) and 45 patients with Class III malocclusion (group CTR, mean age 25.3 ± 5.9). Both groups were skeletally matched based on SNA, SNB, ANB, GoGn‐SN angles and Wits appraisal values. MSV was measured using ITK‐SNAP software. The Kolmogorov–Smirnov test confirmed normal data distribution. Intergroup comparisons of average MSV, MSV stratified per side, cephalometric parameters and gender differences were performed using Student's *t*‐test. Intragroup comparisons (cleft vs. non‐cleft side, right vs. left side) were conducted using a paired *t*‐test. Statistical significance was set at *p* < 0.05.

**Results:**

No statistically significant differences in average MSV were found between the CLP group (7589.74 ± 4060.23 mm^3^) and the CTR group (7901.90 ± 3667.69 mm^3^) (*p* = 0.59). However, within the CLP group, the MSV on the cleft side (6870.53 ± 3695.29 mm^3^) was significantly smaller than on the non‐cleft side (8308.96 ± 4316.53 mm^3^) (*p* < 0.05). No significant gender differences regarding MSV values were found (*p* > 0.05).

**Conclusions:**

Unilateral cleft lip and palate resulted in a significantly smaller MSV on the cleft side in CLP patients. However, the average MSV of CLP patients was not significantly reduced compared to non‐cleft patients with a skeletal Class III pattern.

## 1 Introduction

Cleft lip and palate (CLP) are among the most common congenital developmental anomalies, affecting approximately 1 in 700–1000 live births worldwide [1]. These anomalies arise from the incomplete fusion of the maxillary and medial nasal processes during embryonic development, resulting in a spectrum of defects ranging from cleft lip to more complex forms involving both the lip and palate [2]. Affected individuals face numerous anatomical and functional challenges, including midface hypoplasia, otologic, rhinology and audiological disorders, as well as a heightened susceptibility to maxillary sinusitis [3]. Furthermore, patients with unilateral CLP exhibit anatomical and multidimensional skeletal asymmetries in the frontal, orbital, zygomatic and maxillary regions [4].

The maxillary sinus in CLP patients has garnered significant research interest due to the high prevalence of sinusitis in these patients [3]. Previous studies have attempted to elucidate whether the presence of CLP affects the size and development of the maxillary sinus. The medial nasal process, responsible for developing the primary palate, also influences other midface structures, including the maxilla, lip and nose [5, 6]. This relationship suggests potential anatomical or structural differences between cleft and non‐cleft patients in maxillary sinus development. Moreover, individuals with CLP are often affected by midfacial hypoplasia associated with Class III malocclusion, further complicating the anatomical landscape [7, 8, 9].

Some researchers believe hypoplasia of the maxilla leads to hypoplastic maxillary sinuses [10, 11, 12], which can cause drainage issues due to malpositioned ostia, thereby predisposing patients to sinusitis [3, 13]. Several authors suggest that reduced maxillary sinus volume (MSV) might play a crucial role in the development of sinusitis in CLP patients [14], as they have found either reduced MSV in patients with CLP compared to healthy control groups [11, 12, 15, 16, 17, 18, 19, 20] or reduced MSV on the cleft side compared to the non‐cleft side of CLP patients [15, 16, 21, 22]. Conversely, other researchers found no difference between the MSV of CLP and the non‐cleft group [5, 6, 21] or between the cleft side and non‐cleft side of CLP patients [5, 11, 19, 23, 24] or reported an increased MSV on the cleft side compared to the non‐cleft side [12].

The aetiology of sinusitis in CLP patients is multifactorial and needs to be fully understood. Factors such as velopharyngeal insufficiency, nasal airway obstruction, impaired nasal mucociliary activity, presence of a pharyngeal flap, septal deviation, increased height of the maxillary sinus floor and lateral nasal wall anomalies are also believed to contribute to this condition [3, 13, 25]. It is hypothesised that the lower MSV observed in UCLP patients could result from chronic maxillary sinusitis attacks rather than being directly caused by maxillary hypoplasia due to cleft repair scars [11, 26]. Conversely, some authors have shown that surgical repairs typically impact the cleft region while leaving the posterior maxilla unaffected [27].

The advent of three‐dimensional imaging techniques, particularly cone‐beam computed tomography (CBCT), has revolutionised the assessment of maxillary sinus dimensions. CBCT offers accurate dimensional and volumetric evaluations with minimal radiation exposure, facilitating detailed analysis of sinus morphology in both cleft and non‐cleft individuals [19, 28, 29].

Despite the significant body of research, the literature lacks consensus on the maxillary sinus characteristics in CLP patients [28]. Studies have reported controversial results regarding the maxillary sinus dimensions and volume between CLP patients and healthy individuals [11, 12, 15, 16, 17, 18, 19, 21], as well as between the cleft and non‐cleft sides in unilateral cleft lip and palate (UCLP) patients [11, 15, 16, 19, 21, 22, 23, 24]. Some investigations have shown reduced maxillary sinus dimensions on the cleft side, attributed to skeletal hypoplasia associated with the cleft [15, 16, 21, 22]. However, other studies have not found significant differences in MSV between the cleft and non‐cleft sides [5, 11, 19, 23, 24]. Most of these studies lacked either a control group [23, 24] or included a healthy control group without accounting for the skeletal pattern of these patients [11, 12, 15, 16, 17, 18, 19, 21]. It has been shown that individuals with skeletal Class II malocclusion [30, 31] and high‐angle vertical growth patterns [31], have higher MSV values compared to other growth patterns, while patients with skeletal Class III malocclusion have the lowest MSV values [30]. Considering that CLP, especially in its unilateral form, is frequently associated with skeletal Class III malocclusion, there is a potential bias when the skeletal pattern of the control group is unclear or does not match that of the CLP group [9, 32]. Therefore, the recent literature advises using a control group with a skeletal Class III pattern to isolate the effect of the cleft from skeletal pattern influences, leading to more reliable conclusions [32].

Notably, to our knowledge, no previous study has used a skeletally matched control group to compare MSV in CLP patients. This omission may have contributed to the inconsistent findings in the literature. Therefore, the present study aimed to fill this gap by comparing MSV in patients with right‐sided unilateral nonsyndromic CLP using CBCT images to a skeletally matched control group of patients with skeletal Class III malocclusion.

## 2 Methods and Materials

The present cross‐sectional study received ethical approval from the dental school's Ethics Commission under reference number IR.IAU.DENTAL.REC.1402.091. This study used CBCT images and lateral cephalograms obtained from the oral and maxillofacial radiology department archives between 2021 and 2024. All photos were initially taken for medical purposes unrelated to this study.

The study included two groups: Group CLP (*n* = 45), consisting of patients with right‐sided UCLP and Group CTR (*n* = 45), the control group comprising patients with skeletal Class III malocclusion. Only individuals meeting the following inclusion criteria for each group were included, excluding those who met any specified exclusion criteria.

**FIGURE 1:**
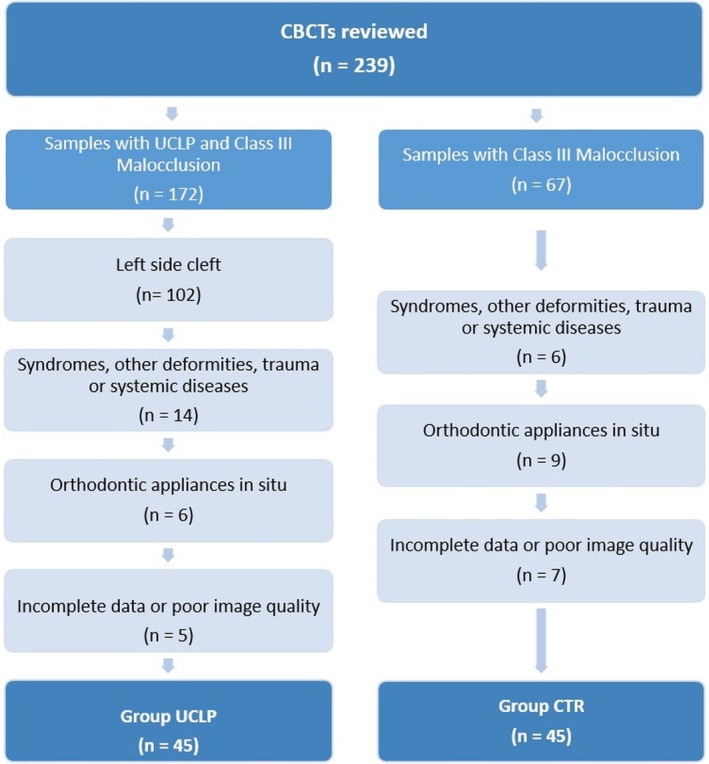
Selection process for inclusion and exclusion of the patients.

The inclusion criteria were patients referred to the orthognathic surgery department of the university aged between 16 and 40 with both lateral cephalograms and high‐quality CBCT images and either UCLP on the right side or skeletal Class III malocclusion [33], with an ANB angle of 0 or below 0 degrees and a Wits value below −1. Patients with previous preoperative orthodontic treatment, orthognathic surgery (other than CLP closure), facial trauma, craniofacial deformities other than CLP, systemic diseases or conditions affecting the size and volume of the maxillary sinus were excluded from the study (Figure 1).

The sample size was calculated using G‐Power (version 3.1.9.2; Heinrich‐Heine‐Universität Düsseldorf, Düsseldorf, Germany). Based on the results of a previous study, an effect size of 0.61 for MSV in the UCLP and control groups was selected [21].

Based on a two‐tailed Student's *t*‐test for independent samples, with an allocation ratio of 1:1, a significance level of 0.05 and a power of 80% to detect an effect size of 0.61, a minimum of 44 patients per group was necessary.

All of the CBCT images were taken under standard conditions, with the patients in maximum intercuspation and an upright position, using the Rotograph Evo 3D CBCT scanner (Villa Sistemi Medicali, Buccinasco MI, Italy) with exposure settings of 85 kVp, 8.5 × 8.5 cm field of view, 9 mA and 185 μm voxel size.

**FIGURE 2:**
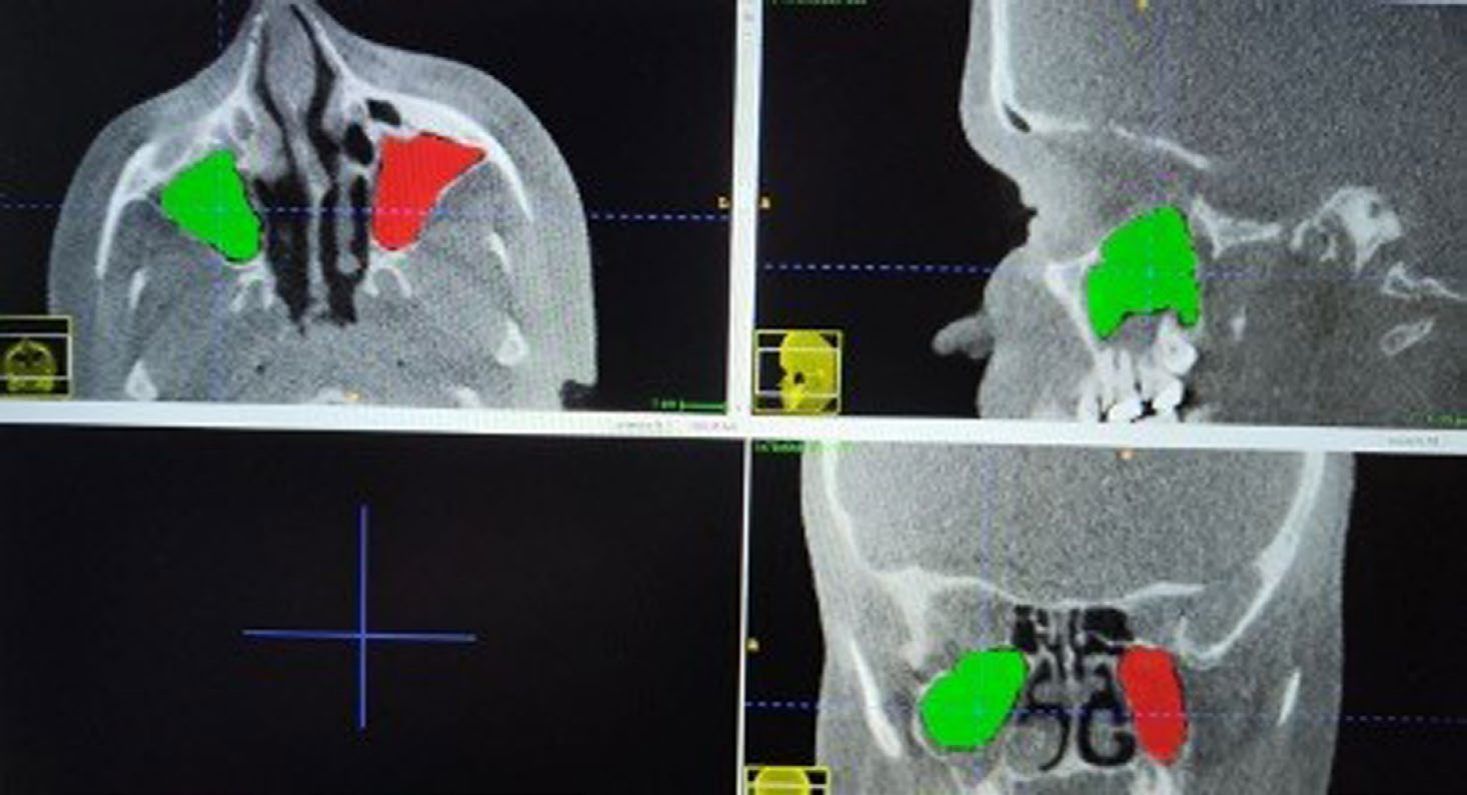
The thresholds of the maxillary sinus in axial, sagittal and coronal planes.

**FIGURE 3:**
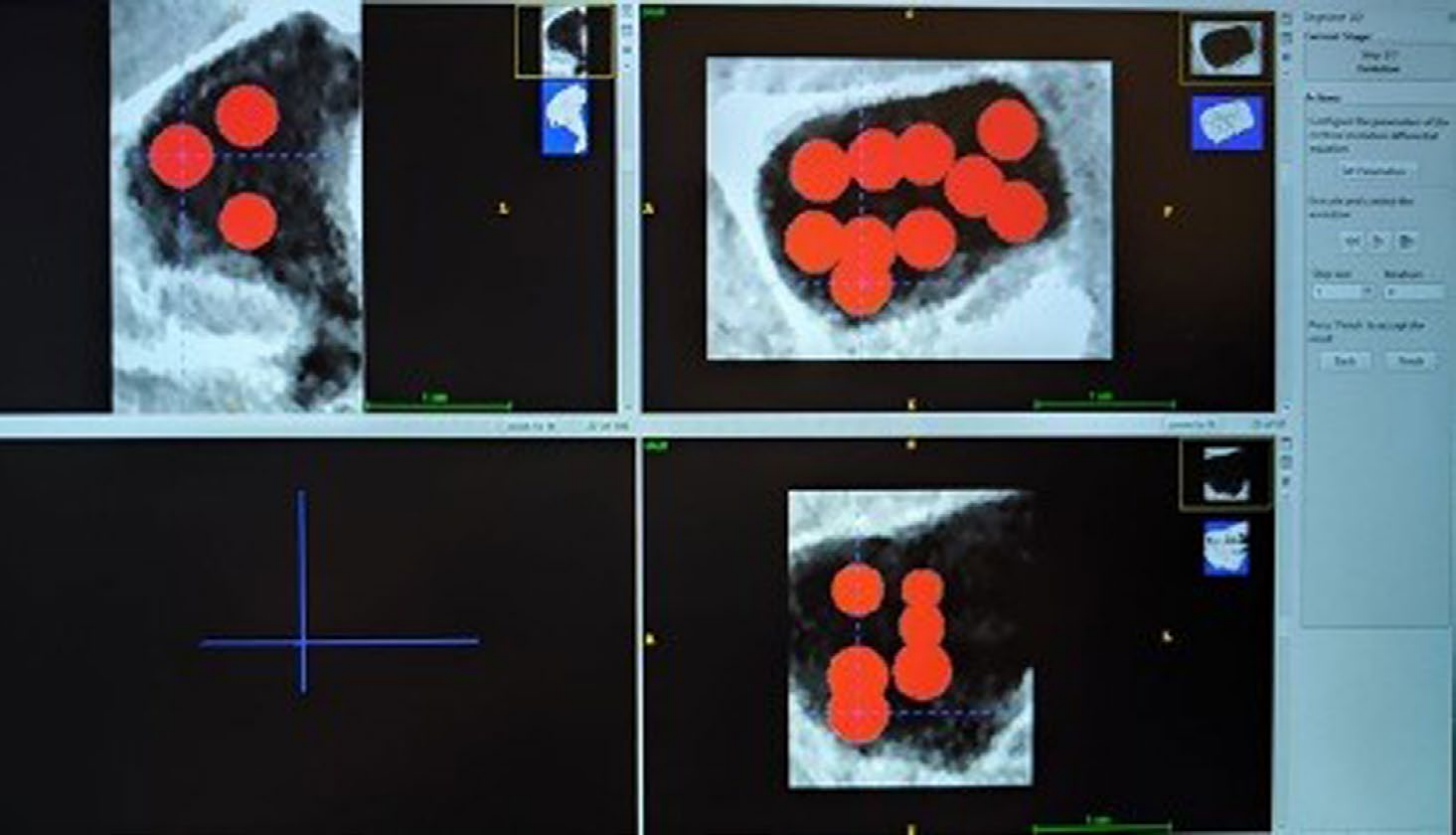
Segmentation process using the ITK‐SNAP software.

The data obtained from the CBCT images were converted to DICOM (Digital Imaging and Communications in Medicine) files, and measurements were conducted using ITK‐SNAP software (version 3.8.0; Cognitica, Philadelphia, PA; at www.itksnap.org) following the methodology outlined by Shrestha et al. [30], while employing a semiautomatic segmentation method. The process began with the right maxillary sinus, where the maximum dimensions were determined in three planes (frontal, lateral and axial) by setting appropriate threshold limits. The thresholded region was then duplicated and the right maxillary sinus was isolated by removing any connections to external air. A 3D reconstruction of the sinus was created and its volume was computed. The same procedure was applied to the left maxillary sinus (Figures 2 and 3).

Additionally, lateral cephalograms were evaluated to measure SNA, SNB, ANB, GoGn–SN angles and Wits appraisal values.

An experienced oral and maxillofacial radiologist and a trained last‐year dental student conducted all measurements. Test–retest reliability was tested by randomly selecting 10 images and repeating the measurements 2 weeks after the initial assessment. The results showed no statistically significant differences between the two sets of measurements (*p* = 0.68).

IBM SPSS software version 25.0 (IBM Corp., Armonk, N.Y., USA) was used to analyse the data. Descriptive statistics were calculated for both groups, including mean, standard deviation and the minimum and maximum dimensions of the maxillary sinus for both the right and left sides. The Kolmogorov–Smirnov test confirmed that the data were normally distributed.

Gender comparisons (female vs. male), as well as intergroup comparative analyses (CLP vs. CTR), were performed using Student's *t*‐test to assess differences in average MSV, MSV stratified per side, SNA, SNB, ANB and GoGn‐SN angles and Wits appraisal. Intragroup comparisons of MSV (right side vs. left side) within each group were conducted using the paired *t*‐test. A significance threshold of *p* < 0.05 was established to determine statistical significance for all tests.

## 3 Results

**FIGURE 4:**
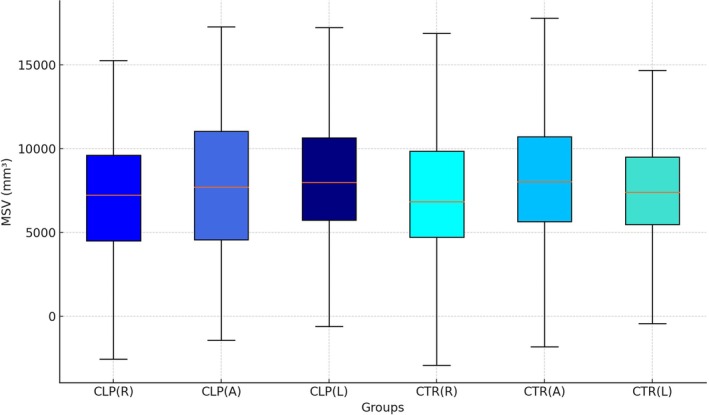
Boxplots representing the MSV values in groups CLP and CTR stratified per side (*R* = right, L = left) and their average value (A = average).

The CBCT images of 90 patients were evaluated in this study, comprising 45 patients with right‐sided UCLP (group CLP, mean age 24.3 ± 6.1) and 45 patients with Class III malocclusion (group CTR, mean age 25.3 ± 5.9). The descriptive analysis of the results is shown in Tables 1 and 2 and Figure 4. Student's *t*‐test revealed no statistically significant differences between CLP and CTR regarding their SNA (*p* = 0.129), SNB (*p* = 0.208), ANB (*p* = 0.845), GoGn‐SN (*p* = 0.724) and Wits appraisal (*p* = 0.237) values (Table 1). Consequently, the patients in both groups were considered homogeneous regarding their skeletal basal relationships of the maxilla and mandible in both the sagittal and vertical dimensions. Group CLP consisted of 25 female and 20 male patients, while group CTR included 26 females and 19 males, indicating homogeneity in gender distribution across both groups.

**TABLE 1:** Comparisons between Group CLP and Group CTR regarding their SNA, SNB, ANB, GoGn‐SN and Wits appraisal values.

Study groups	Variables				
	SNA (Mean ± SD)	SNB (Mean ± SD)	ANB (Mean ± SD)	GoGn‐SN (Mean ± SD)	Wits appraisal (Mean ± SD)
CLP (*n* = 45)	73.5 ± 1.78^a^	77.6 ± 2.32^a^	−4.03 ± 2.13^a^	31.5 ± 1.03^a^	−4.73 ± 2.20 mm
CTR (*n* = 45)	74.1 ± 1.81^a^	78.3 ± 2.82^a^	−4.14 ± 3.10^a^	31.6 ± 0.87^a^	−5.38 ± 2.92 mm
*p*	0.129	0.208	0.845	0.724	0.237

**TABLE 2:** Comparisons between groups CLP and CTR regarding MSV values.

Variable	Study groups	Average MSV (Mean ± SD) (mm^3^)	*p* ^a^	Stratification per side		MSV stratified based on side (Mean ± SD) (mm^3^)	Intergroup comparisons (CLP vs. CTR)	Intragroup comparisons (right vs. left)
							*p* ^b^	*p* ^c^
MSV	CLP	7589.74 ± 4060.23	0.59	CLP	R (cleft)	6870.53 ± 3695.29	0.28	0.001***
					L	8308.96 ± 4316.53	0.72	
	CTR	7901.90 ± 3667.69		CTR	R	7781.93 ± 4031.81	0.28	0.23
					L	8021.87 ± 3305.26	0.72	

Abbreviations: CLP, patients with right‐sided unilateral cleft lip and palate; CTR, patients with class III malocclusion; L, left side; MSV, maxillary sinus volume; *R*, right side (cleft side in CLP group); SD, standard deviation.

^a^ Statistical significance of Student's *t*‐test (CLP vs. CTR) is set at *p* < 0.05.

^b^ Statistical significance of Student's *t*‐test (CLP right vs. CTR right, CLP left vs. CTR left) is set at *p* < 0.05.

^c^ Statistical significance of paired *t* test (CLP right vs. CLP left, CTR right vs. CTR left) is set at *p* < 0.05***.

Comparisons of maxillary sinus volume (MSV) between the right (affected side in the CLP group) and left sides using paired *t*‐tests showed that in the CLP group, MSV on the cleft side (6870.53 ± 3695.29 mm^3^) was significantly less than on the non‐cleft side (8308.96 ± 4316.53 mm^3^) (*p* < 0.05). No statistically significant differences were found between the MSV of the right (7781.93 ± 4031.81 mm^3^) and left sides (8021.87 ± 330.26 mm^3^) in group CTR (*p* = 0.23).

Although the average MSV of group CTR (7901.90 ± 3667.69 mm^3^) was higher than that of group CLP (7589.74 ± 4060.23 mm^3^), no statistically significant differences were identified using Student's *t*‐test (*p* = 0.59). Stratifying the MSV based on the side also yielded similar results, with higher right‐side MSV in group CTR (7781.93 ± 4031.81 mm^3^) compared to group CLP (6870.53 ± 3695.29 mm^3^); however, these differences were not statistically significant (*p* = 0.28). No statistically significant differences were found in the left‐side MSV between the groups either (*p* = 0.72), even though the average MSV on the left (non‐cleft) side was higher in group CLP (8308.96 ± 4316.53 mm^3^) compared to group CTR (8021.87 ± 3305.26 mm^3^).

A further subdivision of the patients in each group was performed based on gender (Table 3). Although males exhibited higher MSV than females on both sides and in both groups, these differences were not statistically significant (*p* > 0.05).

**TABLE 3:** Comparisons between female and male genders in Groups CLP and CTR regarding their MSV values stratified per side.

Study groups	Side	Gender	MSV (Mean ± SD) (mm^3^)	*p* ^a^
CLP	R	F	5830.24 ± 1372.90	0.06
		M	8170.90 ± 5102.72	
	L	F	7903.04 ± 4032.70	0.49
		M	8816.35 ± 4703.05	
CTR	R	F	7665.85 ± 3826.09	0.82
		M	7940.79 ± 4399.73	
	L	F	7978.04 ± 3256.83	0.92
		M	8081.84 ± 3459.26	

Abbreviations: CLP, patients with right sided unilateral cleft lip and palate; CTR, patients with class III malocclusion; F, female; L, left side; M, male; MSV, maxillary sinus volume; *R*, right side (affected side in CLP group); SD, standard deviation.

^a^ Statistical significance of Student's *t* test (F vs. M) is set at *p* < 0.05*.

## 4 Discussion

The present study aimed to measure the maxillary sinus volume (MSV) in patients with UCLP (group CLP) using cone beam computed tomography (CBCT) images and to compare these measurements with a skeletally matched control group of patients with Class III skeletal malocclusion (group CTR). No other study has utilised a skeletally matched control group to compare MSV to this date.

The literature on maxillary sinus volume (MSV) in patients with CLP is highly controversial due to multiple inconsistencies [28]. These arise from varying evaluation methods, such as the use of different 2D [5, 7] and various 3D imaging techniques, such as CT, MRI and CBCT [8, 20, 24, 34], differences in measurement approaches, including volume calculations versus 2D measurements [18, 20, 21, 25] and the use of different software [28]. Other drawbacks include limited sample sizes of [3, 19, 22, 23] and varying age groups [5, 23].

However, the most significant confounding factor is the lack of control groups [22, 23, 24] or the use of controls without considering their skeletal patterns [11, 12, 15, 16, 17, 18, 19, 21]. Control groups with a Class II skeletal growth pattern, known for the highest MSV [30, 31], can skew comparisons with CLP patients, who typically present with a Class III skeletal pattern. This can exaggerate the lower MSV in CLP patients, falsely attributing it to the cleft rather than to skeletal differences.

High‐angle vertical growth patterns are also linked to higher MSV [31], while horizontal growth patterns show the least [35]; including control groups with predominantly horizontal patterns could lead to erroneous conclusions that CLP patients have higher or similar MSV compared to non‐cleft individuals. Thus, skeletal pattern differences in these studies obscure the impact of the cleft, weakening its perceived effect.

The present study ensured the skeletal pattern of both groups (CLP and CTR) was homogenous not only in the sagittal dimension by controlling the SNA, SNB and ANB angles (*p* values: 0.129, 0.208 and 0.845, respectively) but also in the vertical dimension through the GoGn‐SN angle (*p* = 0.724). Additionally, Wits appraisal (CLP = −4.73 ± 2.20 mm, CTR = −5.38 ± 2.92 mm, *p* = 0.237) alongside ANB guaranteed the accurate selection of skeletal Class III cases [36].

Our results showed no statistically significant differences between the MSV of the CLP and CTR groups (*p* = 0.59), even though the average MSV of the CLP group (7589.74 ± 4060.23 mm^3^) was lower than that of the CTR group (7901.90 ± 3667.69 mm^3^). These results are in agreement with findings from previous studies [3, 5, 6, 21, 37]. This finding corroborates the hypothesis of compensatory maxillary sinus enlargement on the non‐cleft side, as evidenced by the significantly higher MSV values on the non‐cleft side (8308.96 ± 4316.53 mm^3^) compared to the cleft side (6870.53 ± 3695.29 mm^3^) in our study (*p* < 0.05), which is also higher than the average MSV of the control group (7901.90 ± 3667.69 mm^3^). These results are also consistent with findings from several previous studies [15, 16, 21, 22].

Both findings of this study align with those of Rodrigues et al. [21], who reported no significant differences in MSV between UCLP patients and controls but found substantial differences between the cleft and non‐cleft sides. While Rodrigues et al. did not use a Class III control group, their large sample size (80 per group) likely mitigated this variable, resulting in comparable findings to our study. Additionally, our use of ITK‐SNAP software (version 3.8.0; Cognitica, Philadelphia, PA) for measurements, similar to Rodrigues et al., may have contributed to the consistency of our results. Despite using a homogeneous sample of patients past their maximal growth peak in our study and detecting higher MSV in males, these differences did not reach statistical significance, contrary to Rodrigues et al. [21], which could be attributed to our smaller sample size. Two separate studies also demonstrated the lack of sexual dimorphism in MSV values [11, 12], highlighting the need for future research with larger sample sizes to improve statistical power.

Contrary to our results, several authors reported significantly smaller MSV in patients with CLP than in healthy individuals [11, 12, 15, 16, 17, 18, 19]. This controversy could be explained by the different structures of the study groups, as these studies all included skeletally matched control samples.

Among these studies, Tunc et al. [15] had the most similar design to ours, and Rodrigues et al. [21] also used ITK‐SNAP software and had a large sample size of patients with UCLP. Similar to our findings, they also showed smaller MSV on the cleft side compared to the non‐cleft side. However, they reported significantly smaller MSV in patients with UCLP compared to their control group. This inconsistency could be attributed to the study participants' potentially wide age range, which could introduce variability in MSV due to differences in sinus development stages and a possible lack of skeletal homogeneity in their control samples.

Contrary to our findings, Altindag et al. [17] documented significantly reduced MSV in their CLP group. However, their cohort included bilateral cleft lip and palate (BCLP) cases and had a limited sample size. Similarly, our study observed diminished MSV on the cleft side, which is exacerbated in BCLP patients [19], as the lack of an unaffected maxillary sinus further accentuates the overall reduction in sinus volume. This highlights the confounding effect of including BCLP samples in the CLP group, thereby reducing the overall MSV in the CLP group.

This confounding factor might explain the significantly smaller MSV reported in CLP patients by several studies. For instance, Altun et al. included 70 UCLP and 30 BCLP cases in their CLP group [16], Paknahad et al. [18] had 40 UCLP and 14 BCLP cases and Barbosa et al. included 30 UCLP and 15 BCLP cases [19]. These inclusions likely contributed to the reduced overall MSV in their CLP groups, supporting the trend observed by Altindag et al. [17].

Contrary to our findings, Erdur et al. [11] documented significantly reduced MSV in CLP patients, with no significant differences between the cleft and non‐cleft sides. This discrepancy can be attributed to methodological differences and the age range of their study participants. Arthur et al.'s cohort (13.8 ± 3.9 years for CLP and 14.2 ± 1.55 years for controls) included pre‐ and post‐peak growth individuals. This inclusion likely introduced significant variability in MSV measurements, as the maturation of the maxillary sinus is closely tied to skeletal and craniofacial development stages [34]. The study by Demirtas et al. [12] used a similar wide age range (13.5 ± 5.0 years for CLP and 14.9 ± 4.2 years for controls), which could explain their controversial results, as they reported higher MSV in CLP patients compared to healthy controls.

The limitations of the present study include its retrospective design and potential heterogeneity within the CLP group concerning their surgical repair procedures. Additionally, the study was conducted at a single centre and involved only one ethnic group, which limits the generalisability of the results.

While this study emphasises sinus volume, recent evidence highlights that airflow efficiency and mucociliary function are primarily governed by morphological features such as ostiomeatal patency, sinus shape and wall orientation [38, 39]. In a 2023 CFD study, Han et al. [38] demonstrated that changing the geometry of the antrostomy site significantly altered ventilation and air‐conditioning characteristics within the sinus, independent of volume changes. Similarly, Warfield‐McAlpine et al. [39] showed that optimising ostium shape rather than enlarging sinus size markedly improved airflow patterns. These findings support a paradigm where functional anatomy rather than static volume is the critical determinant of sinus health. Therefore, future research in cleft‐affected populations should combine volumetric analysis with CFD and anatomical modelling for comprehensive functional assessment [40].

However, the study also has several significant strengths. It effectively isolated the impact of the cleft from skeletal pattern influences by incorporating a skeletally homogeneous sample. Moreover, potential bias was minimised by including only unilateral CLP cases, thereby avoiding the confounding effects of a mixed cohort that provides for bilateral cases.

## 5 Conclusions

The following can be concluded based on the results of the present study:

1. There were no statistically significant differences in MSV between patients with UCLP and those in the skeletal class III control group.
2. MSV of patients with UCLP was significantly smaller on the cleft side compared to the non‐cleft side.

## Author Contributions

**Sara Eslami:** conceptualisation, formal analysis, investigation, writing – original draft, writing – review and editing. **Anand Marya:** software, visualisation. **Babak Sayahpour:** methodology, project administration. **Sarah Bühling:** validation, formal analysis. **Stefan Kopp:** methodology, supervision. **Hanieh Mahmoudi:** software, investigation, data curation. **Ahmadreza Talaeipour:** methodology, investigation, resources. **Ari Harsoputranto:** software, visualisation. **Abdolreza Jamilian:** conceptualisation, methodology, validation, investigation, resources, supervision, writing – review and editing.

## Ethics Statement

The present cross‐sectional study received ethical approval from the Ethics Board of Azad University of Medical Sciences under reference number IR.IAU.DENTAL.REC.1402.091.

## Consent

Informed consent, as well as consent for publication, was obtained from all participants and their legal guardians in the form of broad consent prior to their admission to the university. The authors warrant that the article is original, is not under consideration for publication by another journal, and has not been previously published. The authors approve the publication.

## Conflicts of Interest

The authors declare no conflicts of interest.

## Data Availability

The datasets generated during and/or analysed during the current study are available from the corresponding author upon request.
